# Data-driven mapping of known and novel fluid biomarker progression profiles in genetic frontotemporal lobar degeneration

**DOI:** 10.1186/s13195-026-02159-5

**Published:** 2026-08-11

**Authors:** Julie F. H. De Houwer, Wenjie Kang, Renee van Buuren, Liset de Boer, Tine Swartenbroekx, Ana Rajicic, Arabella Bouzigues, Lucy L. Russell, Phoebe H. Foster, Eve Ferry-Bolder, John C. van Swieten, Lize C. Jiskoot, Raquel Sanchez-Valle, Robert Laforce, Caroline Graff, Daniela Galimberti, Rik Vandenberghe, Alexandre de Mendonça, Pietro Tiraboschi, Isabel Santana, Alexander Gerhard, Johannes Levin, Benedetta Nacmias, Markus Otto, Maxime Bertoux, Thibaud Lebouvier, Simon Ducharme, Chris R. Butler, Isabelle Le Ber, Elizabeth Finger, Maria Carmela Tartaglia, Mario Masellis, James B. Rowe, Matthis Synofzik, Fermin Moreno, Barbara Borroni, Henrik Zetterberg, Yolande A. L. Pijnenburg, Charlotte Teunissen, Jonathan D. Rohrer, Stefan Klein, Elise G. Dopper, Esther E. Bron, Harro Seelaar

**Affiliations:** 1 Department of Neurology and Alzheimer Centre, Erasmus MC University Medical Center, Dr. Molewaterplein 40, Rotterdam, 3015 GD The Netherlands; 2 Biomedical Imaging Group Rotterdam, Department of Radiology & Nuclear Medicine, Erasmus MC University Medical Center, Rotterdam, 3015 GD The Netherlands; 3 Dementia Research Centre, Department of Neurodegenerative Disease, UCL Queen Square Institute of Neurology, University College London, London, WC1N 3AR UK; 4 Alzheimer’s disease and Other Cognitive Disorders Unit, Neurology Service, Institut d’Investigacións Biomèdiques August Pi I Sunyer, Hospital Clínic, University of Barcelona, Barcelona, 08036 Spain; 5 Département des Sciences Neurologiques, Faculté de Médecine, Clinique Interdisciplinaire de Mémoire, CHU de Québec, Université Laval, QC, G1V 4G2 Canada; 6 Department of Neurobiology, Care Sciences and Society, Center for Alzheimer Research, Division of Neurogeriatrics, Karolinska Institutet, Bioclinicum, Solna, 171 65 Sweden; 7 Unit for Hereditary Dementias, Theme Inflammation and Aging, Karolinska University Hospital, Solna, 171 76 Sweden; 8 Department of Biomedical, Surgical and Dental Sciences, University of Milan, Milan, 20122 Italy; 9 Neurodegenerative Diseases Unit, Fondazione IRCCS Ca’ Granda Ospedale Maggiore Policlinico, Milan, 20122 Italy; 10 Laboratory for Cognitive Neurology, Department of Neurosciences, KU Leuven, Leuven, 3000 Belgium; 11 Neurology Service, University Hospitals Leuven, Leuven, 3000 Belgium; 12 Leuven Brain Institute, KU Leuven, Leuven, 3000 Belgium; 13 Faculty of Medicine, University of Lisbon, Lisbon, 1649-028 Portugal; 14 Fondazione IRCCS Istituto Neurologico Carlo Besta, Milano, 20133 Italy; 15 Neurology Service, Faculty of Medicine, University Hospital of Coimbra (HUC), University of Coimbra, Coimbra, 3004-531 Portugal; 16 Center for Neuroscience and Cell Biology, Faculty of Medicine, University of Coimbra, Coimbra, 3004-504 Portugal; 17 Division of Neuroscience and Experimental Psychology, Wolfson Molecular Imaging Centre, University of Manchester, Manchester, UK; 18 Departments of Geriatric Medicine and Nuclear Medicine, University of Duisburg, Essen, Germany; 19 Department of Neurology, Ludwig-Maximilians Universität München, 80802 Munich, Germany; 20 German Center for Neurodegenerative Diseases (DZNE), 81377 Munich, Germany; 21 Munich Cluster of Systems Neurology (SyNergy), 81377 Munich, Germany; 22 Department of Neurofarba, University of Florence, Florence, 50139 Italy; 23 IRCCS Fondazione Don Carlo Gnocchi, Florence, 50139 Italy; 24 Department of Neurology, University of Ulm, Ulm, 45 89081 Germany; 25 Lille Neuroscience & Cognition U1172, University of Lille, CHU Lille, Inserm, 59000 France; 26 Department of Psychiatry, Douglas Mental Health University Institute, McGill University, Montreal, Québec H4H 1R3 Canada; 27 McConnell Brain Imaging Centre, Montreal Neurological Institute, McGill University, Montreal, Québec H4H 1R3 Canada; 28 Nuffield Department of Clinical Neurosciences, Medical Sciences Division, University of Oxford, Oxford, OX3 7JX UK; 29 Department of Brain Sciences, Imperial College London, London, SW7 2AZ UK; 30 Sorbonne Université, Paris Brain Institute – Institut du Cerveau – ICM, Inserm U1127, CNRS UMR 7225, AP-HP - Hôpital Pitié-Salpêtrière, Paris, 75013 France; 31 Centre de référence des démences rares ou précoces, Département de Neurologie, IM2A, AP-HP - Hôpital Pitié-Salpêtrière, Paris, 75013 France; 32 Département de Neurologie, AP-HP - Hôpital Pitié-Salpêtrière, Paris, 75013 France; 33 Department of Clinical Neurological Sciences, University of Western Ontario, London, ON N6A 3K7 Canada; 34 Tanz Centre for Research in Neurodegenerative Diseases, University of Toronto, Toronto, ON M5G 1E8 Canada; 35 Sunnybrook Health Sciences Centre, Sunnybrook Research Institute, University of Toronto, Toronto, ON M4N 3M5 Canada; 36 Department of Clinical Neurosciences, Cambridge University Hospitals NHS Trust, University of Cambridge, Cambridge, CB2 3EB UK; 37 Division of Translational Genomics of Neurodegenerative Diseases, Hertie- Institute for Clinical Brain Research and Center of Neurology, University of Tübingen, 72076 Tübingen, Germany; 38 Center for Neurodegenerative Diseases (DZNE), 72076 Tübingen, Germany; 39 Cognitive Disorders Unit, Department of Neurology, Hospital Universitario Donostia, San Sebastian, 20014 Spain; 40 Neurosciences Area, Group of Neurodegenerative Diseases, Biogipuzkoa Health Research Institute, San Sebastian, 20014 Spain; 41 Center for Biomedical Research in Neurodegenerative Disease (CIBERNED), Carlos III Health Institute, Madrid, 28029 Spain; 42 Department of Clinical and Experimental Sciences, University of Brescia, Brescia, 15-25121 Italy; 43 Molecular Markers Laboratory, IRCCS Istituto Centro San Giovanni di Dio Fatebenefratelli, Brescia, 15-25125 Italy; 44 Department of Psychiatry and Neurochemistry, Institute of Neuroscience and Physiology, Sahlgrenska Academy, University of Gothenburg, Mölndal, 405 30 Sweden; 45 Clinical Neurochemistry Laboratory, Sahlgrenska University Hospital, Mölndal, 413 45 Sweden; 46 Hong Kong Center for Neurodegenerative Diseases, Clear Water Bay, Hong Kong, 999 077 China; 47 Wisconsin Alzheimer’s Disease Research Center, School of Medicine and Public Health, University of Wisconsin, University of Wisconsin-Madison, Madison, WI 53726 USA; 48 Centre for Brain Research, Indian Institute of Science, Bangalore, 560012 India; 49 Amsterdam Neuroscience, Neurodegeneration, Amsterdam, 1081 HV The Netherlands; 50 Alzheimer Center Amsterdam, Amsterdam UMC location VUmc, Vrije Universiteit Amsterdam, Neurology, Amsterdam, 1081 HV The Netherlands; 51 Neurochemistry Laboratory, Department of Laboratory Medicine, Amsterdam Neuroscience, Amsterdam UMC, Vrije Universiteit Amsterdam, Amsterdam, 1081 HV The Netherlands

**Keywords:** Fluid biomarker, Disease progression model, Event-based modelling, Frontotemporal dementia

## Abstract

**Introduction:**

Recent proteomic studies have identified both established and novel proteins in genetic frontotemporal lobar degeneration (FTLD). However, it remains unclear at what point in the disease these proteins deviate from normal levels and how their trajectories relate to one another. Defining the temporal sequence of protein abnormalities could not only improve disease staging but also help identify biomarkers most sensitive to early disease activity in pathogenic variant carriers. We aimed to apply discriminative event-based modelling (DEBM) to characterize the progression profiles of proteins identified in a previous cross-sectional proteomic analysis.

**Methods:**

Building on our prior cross-sectional CSF proteomic analysis of genetic FTLD using a proximity extension assay, we selected the top ten significant proteins for each genetic group (*C9orf72*, *GRN*, *MAPT*). We then applied DEBM to characterize temporal dynamics of these proteins separately in each genetic group and evaluated their potential as early disease markers. To validate model performance, each individual was assigned a disease stage according to their position along the estimated disease timeline, based on protein levels and independent of clinical labels. Next, we assessed how well these stages discriminated symptomatic from presymptomatic carriers and non-carriers.

**Results:**

Across all genetic groups, NfL consistently became abnormal before TPM3, although the earliest abnormal proteins differed between groups. In *C9orf72*, ELAVL4 is the first protein to become abnormal; in *GRN* SEMA3G and *GRN*, and in *MAPT* MMP-10. Estimated individual-level disease stage effectively distinguished symptomatic carriers from presymptomatic carriers and non-carriers, demonstrating high diagnostic accuracy (range AUC 0.74–0.98).

**Conclusion:**

Our data-driven findings provide a temporal ordering of multiple CSF proteins, highlighting potential early biomarkers and disease dynamics in different forms of genetic FTLD. In addition, the model’s accurate estimation of disease stages underscores the value of DEBM for patient stratification, offering a promising tool to support clinical trial design.

**Supplementary Information:**

The online version contains supplementary material available at https://doi.org/10.1186/s13195-026-02159-5.

## Background

Frontotemporal lobar degeneration (FTLD) is a common cause of early-onset dementia and characterized by impairments in behaviour, language and/or motor function [1]. It can be a heritable disorder with a genetic cause in 20–30% of affected individuals [2]. The three most important genes underlying FTLD are microtubule-associated protein tau *(MAPT)*, progranulin *(GRN)* and chromosome 9 open reading frame 72 *(C9orf72)*.

While multiple clinical trials, primarily targeting genetic FTLD, are currently ongoing, there is still no approved disease-modifying treatment. As demonstrated in amyotrophic lateral sclerosis (ALS) [3], the greatest therapeutic benefit can be expected when interventions are initiated early in the disease course, ideally before symptom onset. Importantly, age of disease onset in genetic FTLD shows considerable variability, even among members of the same family [4], indicating that age alone cannot reliably identify individuals approaching symptom onset. Consequently, considerable efforts have focused on identifying cognitive, imaging and fluid biomarkers capable of accurately predicting symptom onset and defining the late presymptomatic stage. Neurofilament light chain (NfL) is one such biomarker and has been shown to be elevated several years before symptom onset [5–7]. However, the longitudinal trajectory of NfL depends on the specific causative gene and phenotype, and its interpretation can be complicated by various factors such as age [8] and renal function [9], highlighting important limitations of NfL as a standalone biomarker.

Therefore, there is a growing interest in the identification of novel fluid biomarkers. One promising approach involves proteomic analyses. Using proximity extension assay technology (Olink^®^) to measure proteins in cerebrospinal fluid (CSF), we previously identified both established and novel proteins associated with FTLD across the three major genetic groups (*C9orf72*, *GRN*, and *MAPT*) [10]. These findings highlighted the involvement of distinct proteins and pathophysiological processes across the different genetic groups. However, it remains unclear when these proteins deviate from normal levels and how their trajectories relate to one another over time. Defining the temporal sequence of protein changes could not only improve disease staging but also help identify markers that are most sensitive to early disease activity in pathogenic variant carriers.

Disease progression models (DPMs) have emerged as valuable approaches to reconstruct the temporal sequence of biomarker changes in neurodegenerative diseases [11]. Collectively, DPMs demonstrate that neurodegenerative diseases are both heterogeneous and temporally complex, and they provide powerful tools to disentangle this complexity and improve patient stratification [12, 13]. In genetic FTLD, these approaches have also proven valuable: subtype and stage inference (SuStaIn) has been used to characterize the clinical features and atrophy patterns of *MAPT*-related FTLD [14] and contrastive trajectory inference has been applied to multi-modal MRI data to derive individualized disease scores in pathogenic variant carriers [15].

Discriminative event-based modelling (DEBM) is a DPM that leverages cross-sectional data to estimate the most likely sequence in which biomarkers become abnormal during the course of disease [16]. Based on their biomarker profiles, individuals can be assigned to a disease stage along this sequence. Importantly, DEBM is robust to missing data and does not rely on predefined diagnostic categories or biomarker cut-off values. We have previously applied DEBM to study cognitive, imaging, and fluid biomarker trajectories in FTLD. In *GRN* carriers, language deficits and serum NfL were among the earliest abnormalities [17], while in a larger genetic cohort, CSF NPTX2 and NfL were the first fluid biomarkers to change [18]. These findings underscore DEBM as a powerful tool for modeling biomarker dynamics in FTLD.

Motivated by the identification of potential gene-specific proteins in our proteomic analysis [10], we aimed to apply DEBM to characterize their progression profiles and evaluate their potential as early markers of disease. Given the known biological heterogeneity across genetic groups, separate models were constructed for *C9orf72*, *GRN*, and *MAPT* carriers to characterize group-specific biomarker progression patterns. Each model included NfL and TPM3, together with additional proteins selected for the respective genetic groups. By reconstructing the temporal sequence of biomarker abnormalities, this approach aims to provide insights into the dynamics of disease progression, with a particular focus on gene-specific trajectories. We hypothesize that the resulting biomarker sequences will reflect the temporal ordering of distinct pathophysiological processes, with markers of synaptic dysfunction becoming abnormal before markers of axonal injury. In *GRN*-related disease, we further expect *GRN* to be among the earliest biomarkers to become abnormal.

## Methods

### Participants

Participants were recruited from 13 centers collaborating in GENFI, a longitudinal cohort study that follows individuals with genetic FTLD due to a pathogenic variant in *GRN*, *C9orf72* or *MAPT* and their 50% at-risk family members (either presymptomatic carriers or non-carriers) [19]. Participants were included if CSF proteome profiles, measured using a proximity extension assay (Olink^®^), were available from our prior cross-sectional study [10]. Participants were classified as symptomatic if they met consensus diagnostic criteria [20–22]. Non-carriers demonstrated normal performance on neuropsychological assessments and did not meet criteria for any neurological or psychiatric disorder. Disease severity was assessed using the CDR Dementia Staging Instrument + National Alzheimer’s Coordinating Centre Frontotemporal Lobar Degeneration component (CDR^®^ + NACC FTLD) sum of boxes. Mini Mental State Examination (MMSE) was used as a screening measure of cognitive function.

### Protein selection

**Fig. 1:**
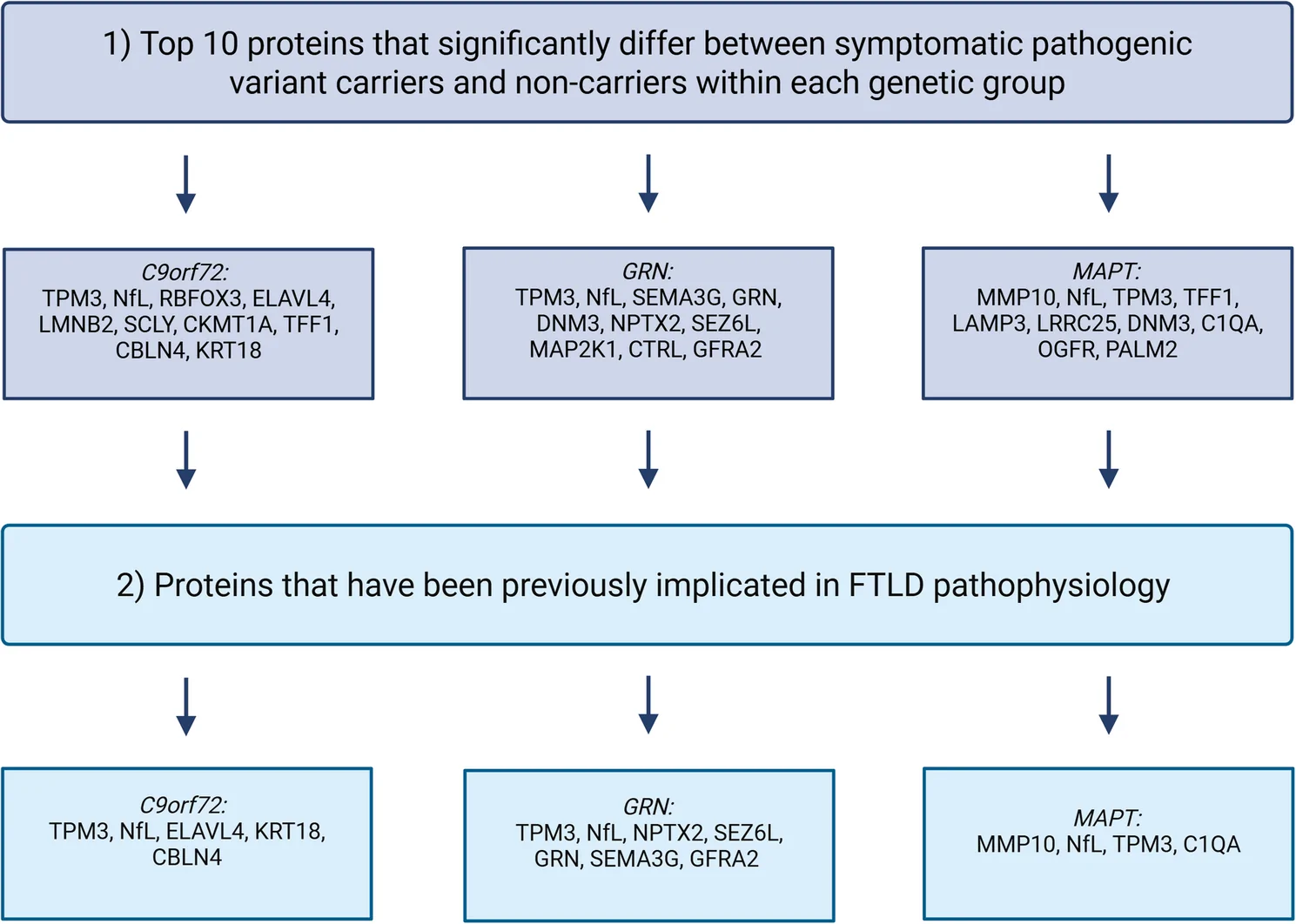
Flowchart displaying the stepwise approach to identify proteins for discriminative event-based modelling

Protein measurements were conducted as part of our previous work, in which over 2,900 proteins were quantified in CSF using proximity extension assay technology [10]. In brief, proteins were measured using the proprietary Olink^®^ Explore 3072 platform. After rigorous data cleaning—retaining proteins that are above the limit of detection in over 85% of samples and passing quality control—1516 proteins were included in subsequent analyses. To assess differences in protein abundance, we constructed linear regression models for each protein, with protein levels as the dependent variable and clinical group as the primary predictor, adjusting for age and sex. This analysis was performed for each genetic group separately. We employed a stepwise approach to identify proteins for further modeling. First, within each genetic group, the top 10 proteins showing significant differences (unadjusted *P* < 0.05) between symptomatic carriers and non-carriers were selected (Fig. 1, Supplementary Table 1, Supplementary Fig. 1, Supplementary Fig. 2). From these candidates, proteins with prior evidence of involvement in FTLD pathophysiology from independent studies were retained. This yielded the following proteins: NfL [23], TPM3 [24], ELAVL4 [25–27], KRT18 [28] and CBLN4 [28, 29] for *C9orf72*; NfL, TPM3, *GRN* [30], NPTX2 [31, 32], SEMA3G [33], SEZ6L [29] and GFRA2 [28, 29] for *GRN*; and NfL, TPM3, MMP10 [28, 34] and C1QA [35] for *MAPT*. For the non-selected proteins, the literature did not provide evidence from independent studies implicating them in FTLD pathophysiology. Therefore, these proteins were not included in the disease progression models, despite their identification in our own proteomics analysis.

### Discriminative event-based modelling

Gene-specific sequences of protein abnormalities and individual-level disease stages were estimated from cross-sectional protein distributions using DEBM [16]. Separate models were constructed for each genetic group (*C9orf72*, *GRN* and *MAPT*), while sharing the same group of non-carrier controls, to account for the known biological heterogeneity and potentially distinct protein progression patterns across genetic groups.

The DEBM modelling consists of three steps: (1) protein abnormality modeling, (2) population-level event ordering and event center estimation, and (3) individual-level disease staging. First, for each protein, normal and abnormal distributions were modelled using two-component Gaussian Mixture Modelling (GMM) [16] fitted to pooled data from non-carriers and carriers (presymptomatic and symptomatic combined). The mixture model has five parameters to be fitted: the mean and standard deviation of the normal component, the mean and standard deviation of the abnormal component, and the mixing proportion. The normal component parameters were fixed to the mean and standard deviation derived from non-carriers, whereas the abnormal component parameters and mixing proportion were estimated from pathogenic variant carriers. The abnormal component was initialized using symptomatic carriers, and the mixing proportion was initialized to 0.5. Components were assigned as normal or abnormal by comparison with empirical group means. Model fit was assessed by visual inspection of stacked histograms with overlaid mixture densities (Supplementary Fig. 3). Second, posterior probabilities of abnormality were computed for each protein in each subject, from which approximate individual-level orderings of protein abnormalities were inferred. These individual sequences were aggregated using a Generalized Mallows model [36] to estimate a population-level ordering of events by minimizing the summed probabilistic Kendall’s Tau distances between the population-level ordering and individual orderings. Proximities of consecutive events in the population-level ordering were estimated based on the cost of swapping adjacent events using probabilistic Kendall’s Tau^16^, resulting in a continuous estimated disease progression trajectory. Third, disease stage was defined as the expected position of an individual along this estimated disease timeline. Following Venkatraghavan et al.^16^, this continuous disease stage was expressed as a continuous score between 0 (all proteins normal) and 1 (all proteins abnormal), independent of clinical labels such as presymptomatic or symptomatic status.

Uncertainty in the estimated ordering was assessed using bootstrap resampling (100 iterations), with the DEBM model refitted in each resampled dataset. Individual-level disease staging and classification performance were evaluated using stratified 10-fold cross-validation within each genetic group. In each fold, mixture model parameters and the population-level ordering were estimated exclusively from the training data and applied to the held-out fold to generate stage-based prediction scores. Classification performance was quantified using balanced accuracy (BA) and area under the receiver operating characteristic curve (AUC) for symptomatic carriers versus non-carriers and symptomatic versus presymptomatic carriers. 95% confidence intervals were estimated using subject-level stratified bootstrap resampling (100 iterations) applied to pooled out-of-fold predictions.

### Statistical analysis

Basic demographics were compared between clinical groups (symptomatic, presymptomatic, non-carrier) with a Kruskal-Wallis test with Dunn’s post-hoc test for continuous variables and a chi-square test for categorical variables. We examined whether estimated disease stages correlated with MMSE and CDR^®^ + NACC FTLD sum of boxes using Spearman’s rank correlations. Differences in clinical variables and relative protein abundance (NPX) between symptomatic carriers with a low estimated disease stage (< 0.5) and with an estimated disease stage ≥ 0.5 were evaluated using the Wilcoxon rank-sum test for continuous variables and the chi-square test for categorical variables.

## Results

### Demographics

In this study, protein measurements were available for 52 symptomatic pathogenic variant carriers (25 *C9orf72*, 17 *GRN* and 10 *MAPT*), 123 presymptomatic pathogenic variant carriers (55 *C9orf72*, 44 *GRN* and 24 *MAPT*), and 80 non-carriers (Table 1). Across all genetic groups, symptomatic carriers were significantly older than both presymptomatic carriers and non-carriers. Additionally, non-carriers were significantly older than presymptomatic *MAPT* carriers. Symptomatic carriers also exhibited significantly lower MMSE scores and higher CDR^®^ + NACC FTLD sum of boxes scores than presymptomatic carriers and non-carriers across all genetic groups. No significant differences in sex distribution were observed among the three clinical groups.

**Table 1:** Subject characteristics

	Symptomatic variant carriers			Presymptomatic variant carriers			Non-carriers	*P*
Per genotype	*C9orf72*	*GRN*	*MAPT*	*C9orf72*	*GRN*	*MAPT*		
*n*	25	17	10	55	44	24	80	
Age at collection, years	65 (46–81)	64 (51–75)	60 (45–68)	43 (29–70)	49 (29–75)	41 (30–68)	46 (31–72)	< 0.001^c^
Sex, female (%)	11 (44%)	8 (47%)	5 (50%)	32 (58%)	20 (45%)	12 (50%)	46 (58%)	> 0.05^d^
MMSE^a^	26 (9–30)	21 (8–28)	27 (16–30)	30 (27–30)	30 (23–30)	30 (27–30)	30 (25–30)	< 0.001^e^
CDR^®^ + NACC FTLD-SB^b^	11 (1–18)	11 (3–18)	9 (2.5–14.5)	0 (0–2.5)	0 (0–4)	0 (0–2.5)	0 (0–5.5)	< 0.001^f^
Phenotype	21 bvFTD 4 FTD-ALS	9 bvFTD 5 nfvPPA 2 PPA-NOS 1 Dementia-NOS	9 bvFTD 1 svPPA	-	-	-	-	-

Continuous variables are expressed as median (range) and were compared between groups using Kruskal-Wallis tests. Categorical variables were compared between groups using Chi-square tests

*Abbreviations*: *MMSE* Mini Mental State Examination, *CDR*^®^ *plus NACC FTLD-SB* CDR Dementia Staging Instrument plus National Alzheimer’s Coordinating Center Frontotemporal Lobar Degeneration component Sum of Boxes, *C9orf72*, chromosome 9 open reading frame 72; *GRN* Progranulin, *MAPT* Microtubule-associated protein tau, *bvFTD* behavioural frontotemporal dementia, *nfvPPA* non-fluent variant primary progressive aphasia, *FTD-ALS* FTD with amyotrophic lateral sclerosis, *PPA-NOS* PPA not otherwise specified dementia-NOS, dementia not otherwise specified, *svPPA* semantic variant PPA

^a^ MMSE data was available for 246 participants

^b^ CDR^®^ plus NACC FTLD-SB data was available for 192 participants

^c^ Symptomatic variant carriers were significantly older compared to presymptomatic variant carriers and non-carriers in all genetic subgroups; in *MAPT* non-carriers were significantly older than non-carriers

^d^ There was no difference in sex distribution between non-carriers, symptomatic and presymptomatic carriers in all genetic subgroups

^e^ MMSE was significantly lower in symptomatic carriers compared to presymptomatic carriers and non-carriers in all genetic subgroups

^f^ CDR^®^ plus NACC FTLD-SB was significantly lower in symptomatic carriers compared to presymptomatic carriers and non-carriers in all genetic subgroups

### Estimation of protein ordering

**Fig. 2:**
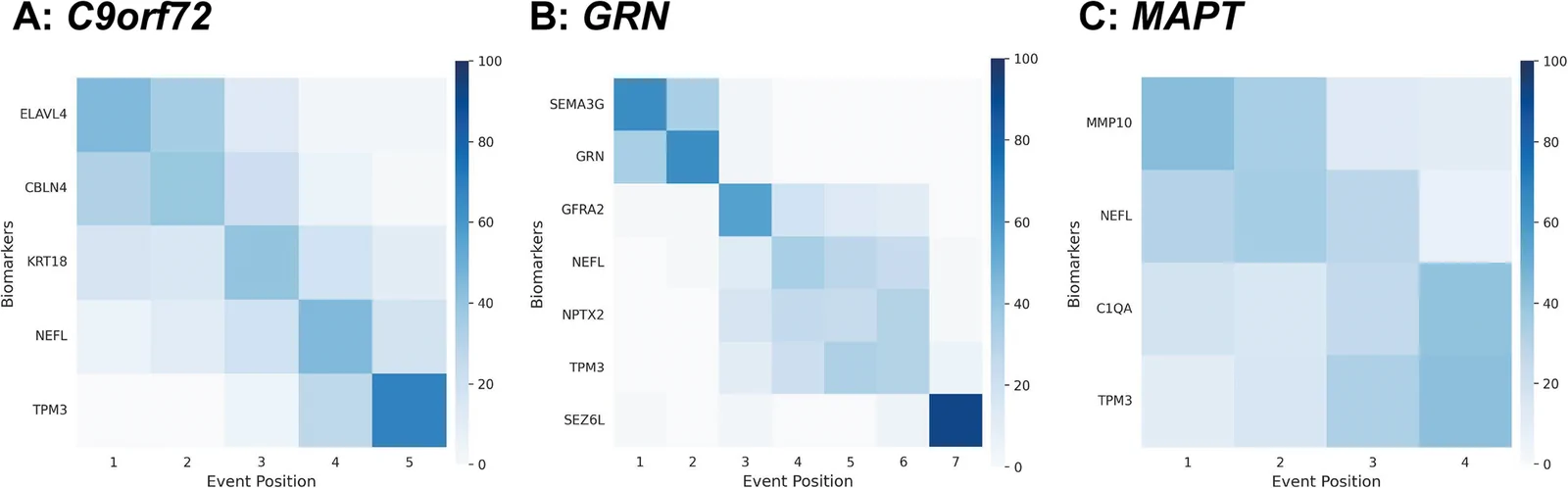
Estimation of population-level protein orderings in the three genetic groups. The positional variance diagrams illustrate the uncertainty in the event ordering, quantified through 100 bootstraps. The color intensity of each square represents the number of bootstrap samples in which a given protein was assigned to a specific position. **A** *C9orf72*, (**B**) *GRN*, (**C**) *MAPT*

Figure 2 shows the estimated population-level ordering of proteins for each genetic group along with their positional variance. In the *C9orf72* group (Fig. 2A), ELAVL4 showed the highest probability of occupying the earliest event position, followed by CBLN4. TPM3 had the highest probability of occurring at the latest position, while KRT18 and NfL exhibited overlapping positional probability distributions, suggesting that they become abnormal in close succession. For *GRN* carriers (Fig. 2B), SEMA3G and *GRN* were most likely to occur at the earliest positions, whereas SEZ6L was most frequently assigned to the latest position. The remaining proteins showed greater positional uncertainty, with broader probability distributions across multiple positions. Among *MAPT* carriers (Fig. 2C), MMP10 most frequently occupied the earliest position, followed by NfL and C1QA, which displayed substantial overlap in their positional distributions. TPM3 again showed the highest probability of being among the last events. Across all genetic groups, NfL and TPM3 were included in the modeling. NfL generally showed peak probability in the middle of the sequence, whereas TPM3 consistently exhibited a high probability of being ranked among the final two events. Supplementary Fig. 3 shows histograms of the selected proteins alongside GMM-estimated normal and abnormal Gaussian distributions for each protein. The inferred protein ordering and estimated event centers formed the basis for estimating individual-level continuous disease stages. Event-center plots illustrating the estimated progression structure are presented in Supplementary Fig. 4.

### Estimation of disease stage

**Fig. 3:**
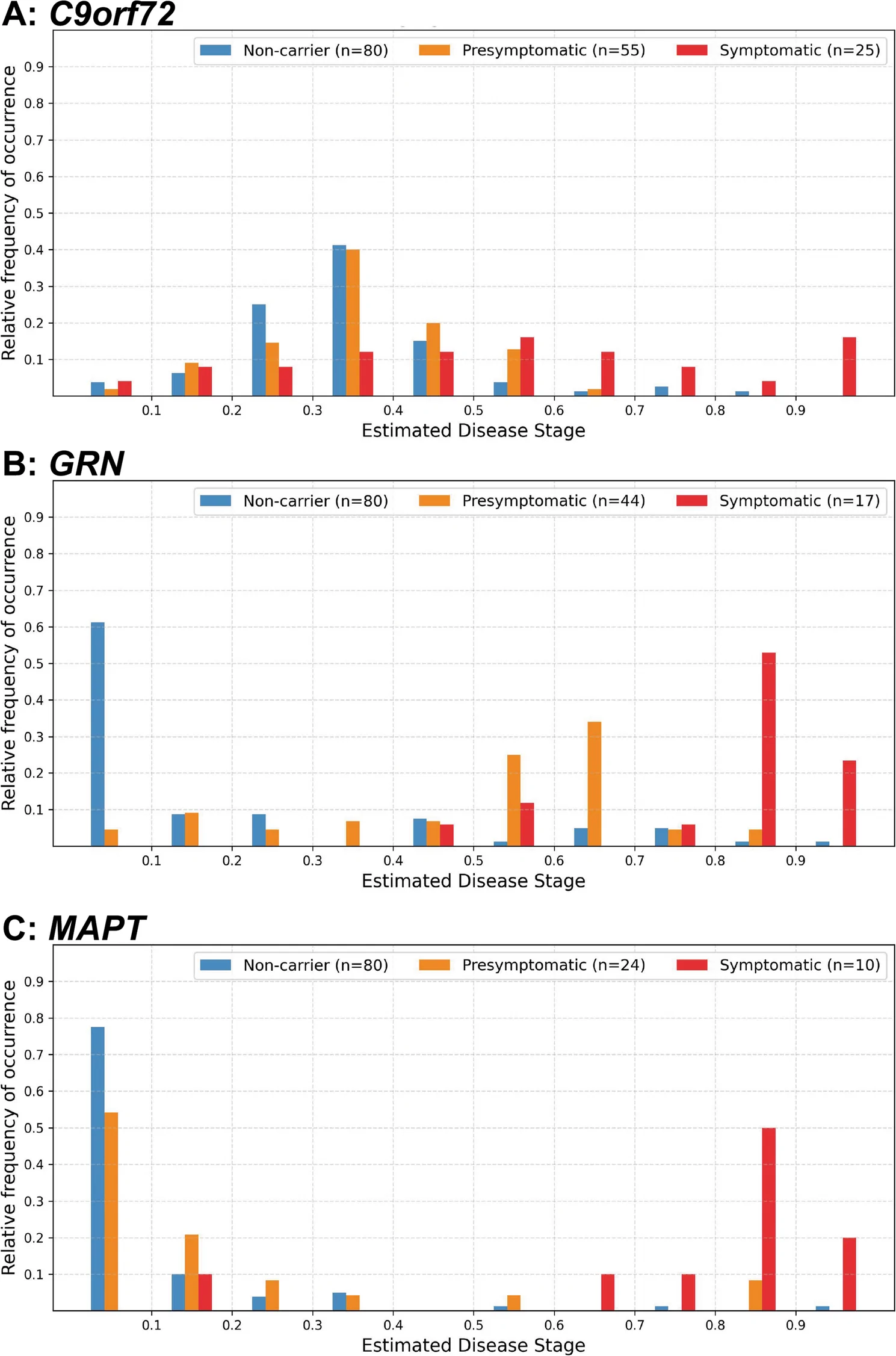
Estimated individual-level disease stages in the three genetic groups. Disease stages were obtained using 10-fold cross-validation. Estimated disease stages are continuous measures and were discretized into 10 bins for visualization purposes only. The histograms show the distribution of subjects in relative frequency across disease stages, normalized within each clinical group. **A** *C9orf72*, (**B**) *GRN*, (**C**) *MAPT*

Based on the inferred protein ordering and estimated event centers, continuous individual-level disease stages ranging from 0 to 1 were assigned to each subject. Across all genetic groups, estimated individual-level disease stage effectively distinguished symptomatic carriers from presymptomatic carriers and non-carriers, demonstrating high diagnostic accuracy (Table 2). However, classification performance in the *C9orf72* group (symptomatic carrier vs. non-carrier: AUC = 0.77 [95%CI: 0.62–0.87]; symptomatic vs. presymptomatic carrier: AUC = 0.74 [0.61–0.89]) was lower than that observed in the *GRN* group (symptomatic carrier vs. non-carrier: AUC = 0.96 [0.91–1]; symptomatic vs. presymptomatic carrier: AUC = 0.89 [0.81–1]) and *MAPT* group (symptomatic carrier vs. non-carrier: AUC = 0.98 [0.94–1]; symptomatic vs. presymptomatic carrier: AUC = 0.93 [0.86–1]) groups. Consistent with the overall diagnostic accuracy, most non-carriers and presymptomatic carriers were assigned to lower disease stages, whereas symptomatic carriers generally clustered at higher stages (Fig. 3). An exception was observed in the *C9orf72* group (Fig. 3A), in which symptomatic carriers were often classified at lower disease stages. To explore this pattern, we stratified symptomatic carriers into two groups based on estimated disease stage: low stage (< 0.5) and high stage (≥ 0.5) (Table 3). Among symptomatic *C9orf72* carriers, 11 individuals were estimated to be in the low disease-stage group and 14 in the high disease-stage group. We did not observe any statistically significant differences between these two groups regarding age at collection, sex, MMSE, CDR^®^ + NACC FTLD sum of boxes or disease duration. Comparison of relative protein abundance revealed lower levels of NfL and TPM3 in the low disease-stage group (Supplemental Fig. 5), whereas the other three proteins (ELAVL4, CBLN4, KRT18) showed no significant differences. For both *GRN* and *MAPT*, only one symptomatic carrier was assigned to a low disease stage, precluding statistical comparisons.

**Table 2:** Classification performance for each genetic subgroup and task

Genetic Subgroup	Task	BA [CI]	AUC [CI]
*C9orf72*	symptomatic vs. non-carrier	0.736 [0.641–0.832]	0.765 [0.616–0.866]
	symptomatic vs. presymptomatic	0.707 [0.618–0.837]	0.737 [0.606–0.885]
*GRN*	symptomatic vs. non-carrier	0.902 [0.815–0.950]	0.960 [0.911–0.995]
	symptomatic vs. presymptomatic	0.630 [0.557–0.716]	0.889 [0.811–0.997]
*MAPT*	symptomatic vs. non-carrier	0.931 [0.803–0.991]	0.976 [0.941–1.00]
	symptomatic vs. presymptomatic	0.887 [0.785–0.979]	0.925 [0.858–1.00]

*Abbreviations*: *C9orf72* Chromosome 9 open reading frame 72, *GRN* Progranulin, *MAPT* Microtubule-associated protein tau, *BA* Balanced accuracy, *AUC* Area under the curve

**Table 3:** Clinical characteristics of symptomatic variant carriers with an estimated disease stage < 0.5 and ≥ 0.5

	Estimated disease stage < 0.5			Estimated disease stage ≥ 0.5			*P* ^c^
	*C9orf72*	*GRN*	*MAPT*	*C9orf72*	*GRN*	*MAPT*	*C9orf72*
*n*	11	1	1	14	16	9	-
Age at collection, years	58 (46–74)	71	58	66 (51–81)	64 (51–75)	62 (45–68)	0.38
Sex, female (%)	6 (55%)	1 (100%)	1 (100%)	5 (36%)	7 (44%)	4 (44%)	0.346
MMSE^a^	26 (14–30)	19	30	26 (9–30)	21 (8–28)	26 (16–30)	0.484
CDR^®^ + NACC FTLD-SB^b^	11 (2.5–18)	17	2.5	11 (1–18)	9 (3–18)	10 (3.5–14.5)	0.675
Disease duration	5 (2–15)	13	2	4 (0–22)	2 (0–8)	3 (0–10)	0.31
Phenotype	11 bvFTD	1 bvFTD	1 bvFTD	10 bvFTD 4 FTD-ALS	8 bvFTD 5 nfvPPA 2 PPA-NOS 1 Dementia-NOS	8 bvFTD 1 svPPA	-

Continuous variables are expressed as median (range) and were compared between groups using a Wilcoxon rank-sum test. Categorical variables were compared between groups using Chi-square test

*Abbreviations*: *MMSE* Mini Mental State Examination, *CDR*^®^ *plus NACC FTLD-SB* CDR Dementia Staging Instrument plus National Alzheimer’s Coordinating Center Frontotemporal Lobar Degeneration component Sum of Boxes, *C9orf72* Chromosome 9 open reading frame 72, GRNP Progranulin, *MAPT* Microtubule-associated protein tau, *bvFTD* behavioural frontotemporal dementia, *nfvPPA* non-fluent variant primary progressive aphasia, *FTD-ALS* FTD with amyotrophic lateral sclerosis, *PPA-NOS* PPA not otherwise specified, *dementia-NOS* dementia not otherwise specified, *svPPA* semantic variant PPA;

^a^ MMSE data was available for 51 participants

^b^ CDR^®^ plus NACC FTLD-SB data was available for 37 participants

^c^ As only 1 participant had a low estimated disease stage in the *GRN* and *MAPT* subgroup, statistical comparison was not possible. *P*-values represent the comparison in the *C9orf72* subgroup

We next examined the relationship between estimated individual-level disease stage and measures of disease severity. Estimated disease stage was negatively correlated with MMSE scores across all genetic groups (*C9orf72*: r_s_ = -0.28, *P* = 0.016; *GRN*: r_s_ = -0.31, *P* = 0.013; *MAPT*: r_s_ = -0.55, *P* < 0.001; Supplemental Fig. 6). In addition, estimated disease stage was positively correlated with CDR^®^ + NACC FTLD sum of boxes in the *C9orf72* and *MAPT* groups, whereas no significant correlation was observed in the *GRN* group (*C9orf72*: r_s_ = 0.29, *P* = 0.016; *GRN*: r_s_ = 0.32, *P* = 0.057; *MAPT*: r_s_ = 0.70, *P* < 0.001; Supplemental Fig. 7).

## Discussion

In this study, we developed a disease progression timeline for the three main genetic groups of FTLD (i.e. *C9orf72*, *GRN* and *MAPT*) using CSF proteins identified in a proteomics analysis [10]. We incorporated both non-specific markers of neurodegeneration and potential gene-specific markers. Across all groups, NfL consistently changed before TPM3. In addition, each model identified potential early gene-specific biomarkers, including ELAVL4 for *C9orf72*, *GRN* and SEMA3G for *GRN*, and MMP10 for *MAPT*. Finally, the model’s ability to accurately estimate disease stages, especially in *GRN* and *MAPT* carriers, highlights its value for patient stratification, offering a promising tool to support clinical trial design.

NfL and TPM3 were included in all models and exhibit a consistent sequence across genetic groups. TPM3, an actin-associated protein, regulates the structure and dynamics of actin filaments [37]. Although primarily expressed in muscle tissue [37], emerging evidence points to a key role for TPM3 in the postsynaptic actin cytoskeleton within the central nervous system [38, 39]. Its involvement in neurodegenerative diseases, however, remains underexplored. The observed sequence of NfL preceding TPM3 abnormalities mirrors previous findings on serum NfL and phosphorylated neurofilament heavy (pNfH) [7]. That study indicated that the onset of symptoms is marked by elevated NfL levels while pNfH remains normal, with pNfH rising later as symptoms progress. Similarly, measuring NfL and TPM3 together might help in disease staging, suggesting that early-stage FTLD could be characterized by elevated NfL but normal TPM3 levels. Moreover, the consistent late position of both NfL and TPM3 suggests that, although distinct pathophysiological processes may predominate early in the disease across genetic groups, they ultimately converge as neurodegeneration progresses, with markers of axonal injury becoming increasingly prominent. It should be noted, however, that because separate models were constructed for each genetic group using different sets of proteins, the relative positions of NfL and TPM3 cannot be directly compared across groups.

For *C9orf72*, the model identified ELAVL4 and CBLN4 as the earliest proteins to become abnormal. ELAVL4, a member of the Hu protein family, is an RNA-binding protein involved in the regulation of RNA metabolism [40, 41]. A recent network analysis emphasized the importance of RNA splicing dysfunction in FTLD too, having observed it across all genetic groups, but most pronounced in *C9orf72* [28]. Our findings support this and suggest that ELAVL4 and similar proteins could play a role early in the disease course. In contrast, CBLN4 is known to be involved in synaptic plasticity [42–44]. This aligns with our previous findings using DEBM, where NPTX2, another synaptic protein, was the first to show abnormality in genetic FTLD [18]. Taken together, these findings support the notion that synaptic dysfunction is an early feature of neurodegeneration in FTLD [45]. While CBLN4 was included only in the *C9orf72*-specific modeling in this study, our prior work identified it as the top hit in a TDP-pathology sub analysis [10]. However, a separate proteomic study observed lower CBLN4 levels across all three genetic groups [28]. These findings highlight the potential of CBLN4 as an early biomarker for FTLD, though further research is needed to clarify its specificity and assess whether it offers advantages over established synaptic markers such as the neuronal pentraxins.

In *GRN*-related FTLD, SEMA3G and *GRN* were the earliest proteins to become abnormal, followed by a broader overlap of the other proteins. Pathogenic variants in *GRN* cause haploinsufficiency, leading to reduced *GRN* levels early in life [30], the close temporal positioning of SEMA3G and *GRN* in our model may suggest that SEMA3G alterations also occur relatively early in the disease process. As a member of the semaphorin family, SEMA3G is part of a group of cell guidance cue proteins involved in cellular differentiation and migration [46]. It is secreted by endothelial cells and may function as a vascular-derived synaptic organizer [47]. Moreover, our postmortem study using single-cell RNA sequencing of human brain tissue [33] underscores the importance of the neurovascular unit in the pathophysiology of *GRN*-related disease. Understanding the interplay between *GRN *haploinsufficiency and reduced SEMA3G levels, and how this contributes to neurovascular dysfunction, warrants further investigation. Aside from SEMA3G and *GRN*, the ordering of protein shows substantial overlap. Notably, in contrast to our previous findings [18], NPTX2 does not clearly precede NfL in this model. This discrepancy may be due to the smaller sample size in the current study and the focus on *GRN* instead of the whole FTLD spectrum.

In *MAPT*, there is significant overlap between the four proteins in this model, with all of them placed relatively late in the ordering, as illustrated by the event center variance diagram. MMP-10 was modeled to become abnormal first. As a member of the matrix metalloproteinase family, MMP-10 is a zinc-dependent protease involved in processes like extracellular matrix remodeling and neuroinflammation [48]. In Alzheimer’s disease, elevated CSF levels of MMP-10 have been consistently reported, with a clear correlation to disease severity [49–51]. Further investigations are required to elucidate the relationship between MMP-10 and disease severity, though its position in this ordering suggests a similar relationship in *MAPT*-related disease.

To validate the models, we showed that the estimated disease stages reliably differentiated symptomatic carriers from presymptomatic carriers and non-carriers, and significantly correlated with MMSE and CDR^®^ + NACC FTLD sum of boxes scores. The high diagnostic accuracy observed indicates that these models are promising tools for disease staging. However, within the *C9orf72* group, some symptomatic carriers were assigned relatively low disease stages, leading to lower diagnostic accuracy than that for the *GRN* and *MAPT* groups. To explore this further, we compared symptomatic *C9orf72* carriers with a low estimated disease stage (< 0.5) to those with a higher disease stage (≥ 0.5). We hypothesized that symptomatic carriers with lower disease stages would exhibit higher MMSE scores, lower CDR^®^ + NACC FTLD sum of boxes scores, or longer disease duration, reflecting a more indolent disease course. This would be consistent with the well-documented heterogeneity and often slow progression of *C9orf72*-related disease, which may span several decades and be reflected in less pronounced protein level changes [4, 52]. Yet, we observed no significant differences in these measures of clinical severity between symptomatic carriers with low versus high disease stages. Individuals with a low disease stage have lower levels of NfL and TPM3, consistent with the model assigning earlier stages to individuals with less pronounced biomarker abnormalities and contributing to the lower diagnostic accuracy observed compared with *GRN* and *MAPT*. Another potential contributing factor, which we did not account for in this study, is the influence of known genetic [53, 54] and epigenetic [55] modifiers that may contribute to the heterogeneity and lower diagnostic accuracy observed in *C9orf72*-related disease. It is also possible that, since disease stages are calculated based on the average estimated sequence of biomarker changes across the study population, individuals who deviate from this typical sequence may be misclassified. In such cases, other data-driven subtyping approaches—such as SuStaIn [12]—could potentially identify groups of individuals with distinct patterns of disease progression.

A key strength of this study is the inclusion of novel proteins—previously unstudied in the context of FTLD or neurodegeneration—identified through proteomic analysis [10]. This approach allowed us to explore new candidate biomarkers for FTLD and highlight their potential relevance for future research and biomarker development. Another strength lies in the use of DEBM, an approach that has the ability to construct a disease progression timeline using cross-sectional data. This is particularly valuable in the context of a rare disease like FTLD, for which longitudinal data can be limited. However, several limitations should be considered when interpreting our findings. First, this analysis was conducted within a single cohort, and protein levels were measured using a proximity extension assay, which provides relative rather than absolute concentrations. As a result, the generalizability of these findings to other populations or platforms has yet to be demonstrated. Second, separate modeling within each genetic group was performed to accommodate biological heterogeneity and distinct protein profiles across genetic groups. However, this resulted in relatively small group sample sizes, particularly in the *MAPT* group, which may reduce the robustness and stability of the estimated ordering. Consequently, the number of proteins that could be included in each model was limited, requiring us to select a subset of candidate biomarkers for each genetic group. As a result, we cannot exclude the possibility that additional proteins may also represent early changes or occupy different positions within the temporal sequence. Third, as with all event-based models based on cross-sectional data, DEBM estimates the temporal ordering of biomarker changes relative to one another but does not establish whether these changes occur before or after symptom onset. Longitudinal data will be important to validate the inferred trajectories and to better anchor the event ordering to clinically meaningful time points. Finally, the representation of individuals at the extreme ends of the disease spectrum (particularly those in advanced stages) is limited in our cohort, which may influence the estimated ordering toward earlier stages.

In conclusion, this study provides an informative event ordering of multiple CSF proteins across distinct genetic groups of FTLD. The accurate estimation of disease stages, particularly in *GRN* and *MAPT*, highlights the model’s potential as a tool for patient stratification.

## Supplementary Information

Supplementary Material 1.

## Data Availability

Data can be accessed upon reasonable request to the corresponding author.

## Appendix

GENFI consortium list

**Table:**

Rhian Convery	Department of Neurodegenerative Disease, Dementia Research Centre, UCL Queen Square Institute of Neurology, London, UK
Martina Bocchetta	Department of Neurodegenerative Disease, Dementia Research Centre, UCL Queen Square Institute of Neurology, London, UK
David Cash	Department of Neurodegenerative Disease, Dementia Research Centre, UCL Queen Square Institute of Neurology, London, UK
Sophie Goldsmith	Department of Neurodegenerative Disease, Dementia Research Centre, UCL Queen Square Institute of Neurology, London, UK
Kiran Samra	Department of Neurodegenerative Disease, Dementia Research Centre, UCL Queen Square Institute of Neurology, London, UK
David L. Thomas	Neuroimaging Analysis Centre, Department of Brain Repair and Rehabilitation, UCL Institute of Neurology, Queen Square, London, UK
Thomas Cope	Cambridge University Hospitals NHS Trust, Cambridge UK
Maura Malpetti	Department of Clinical Neurosciences, University of Cambridge, Cambridge, UK
Antonella Alberici	Centre for Neurodegenerative Disorders, Department of Clinical and Experimental Sciences, University of Brescia, Brescia, Italy
Enrico Premi	Stroke Unit, ASST Brescia Hospital, Brescia, Italy
Roberto Gasparotti	Neuroradiology Unit, University of Brescia, Brescia, Italy
Emanuele Buratti	ICGEB Trieste, Italy
Valentina Cantoni	Centre for Neurodegenerative Disorders, Department of Clinical and Experimental Sciences, University of Brescia, Brescia, Italy
Andrea Arighi	Fondazione IRCCS Ca’ Granda Ospedale Maggiore Policlinico, Neurodegenerative Diseases Unit, Milan, Italy
Chiara Fenoglio	University of Milan, Centro Dino Ferrari, Milan, Italy
Vittoria Borracci	Fondazione IRCCS Ca’ Granda Ospedale Maggiore Policlinico, Neurodegenerative Diseases Unit, Milan, Italy
Maria Serpente	Fondazione IRCCS Ca’ Granda Ospedale Maggiore Policlinico, Neurodegenerative Diseases Unit, Milan, Italy
Tiziana Carandini	Fondazione IRCCS Ca’ Granda Ospedale Maggiore Policlinico, Neurodegenerative Diseases Unit, Milan, Italy
Emanuela Rotondo	Fondazione IRCCS Ca’ Granda Ospedale Maggiore Policlinico, Neurodegenerative Diseases Unit, Milan, Italy
Giacomina Rossi	Fondazione IRCCS Istituto Neurologico Carlo Besta, Milano, Italy
Giorgio Giaccone	Fondazione IRCCS Istituto Neurologico Carlo Besta, Milano, Italy
Giuseppe Di Fede	Fondazione IRCCS Istituto Neurologico Carlo Besta, Milano, Italy
Paola Caroppo	Fondazione IRCCS Istituto Neurologico Carlo Besta, Milano, Italy
Sara Prioni	Fondazione IRCCS Istituto Neurologico Carlo Besta, Milano, Italy
Veronica Redaelli	Fondazione IRCCS Istituto Neurologico Carlo Besta, Milano, Italy
David Tang-Wai	The University Health Network, Krembil Research Institute, Toronto, Canada
Ekaterina Rogaeva	Tanz Centre for Research in Neurodegenerative Diseases, University of Toronto, Toronto, Canada
Miguel Castelo-Branco	Faculty of Medicine, ICNAS, CIBIT, University of Coimbra, Coimbra, Portugal.
Morris Freedman	Baycrest Health Sciences, Rotman Research Institute, University of Toronto, Toronto, Canada
Ron Keren	The University Health Network, Toronto Rehabilitation Institute, Toronto, Canada
Sandra Black	Sunnybrook Health Sciences Centre, Sunnybrook Research Institute, University of Toronto, Toronto, Canada
Sara Mitchell	Sunnybrook Health Sciences Centre, Sunnybrook Research Institute, University of Toronto, Toronto, Canada
Christen Shoesmith	Department of Clinical Neurological Sciences, University of Western Ontario, London, Ontario, Canada
Robart Bartha	Department of Medical Biophysics, The University of Western Ontario, London, Ontario, Canada
Rosa Rademakers	Center for Molecular Neurology, University of Antwerp
Jackie Poos	Department of Neurology, Erasmus Medical Center, Rotterdam, Netherlands
Janne M. Papma	Department of Neurology, Erasmus Medical Center, Rotterdam, Netherlands
Lucia Giannini	Department of Neurology, Erasmus Medical Center, Rotterdam, Netherlands
Liset de Boer	Department of Neurology, Erasmus Medical Center, Rotterdam, Netherlands
Julie De Houwer	Department of Neurology, Erasmus Medical Center, Rotterdam, Netherlands
Rick van Minkelen	Department of Clinical Genetics, Erasmus Medical Center, Rotterdam, Netherlands
Yolande Pijnenburg	Amsterdam University Medical Centre, Amsterdam VUMC, Amsterdam, Netherlands
Benedetta Nacmias	Department of Neuroscience, Psychology, Drug Research and Child Health, University of Florence, Florence, Italy
Camilla Ferrari	Department of Neuroscience, Psychology, Drug Research and Child Health, University of Florence, Florence, Italy
Cristina Polito	Department of Biomedical, Experimental and Clinical Sciences “Mario Serio”, Nuclear Medicine Unit, University of Florence, Florence, Italy
Gemma Lombardi	Department of Neuroscience, Psychology, Drug Research and Child Health, University of Florence, Florence, Italy
Valentina Bessi	Department of Neuroscience, Psychology, Drug Research and Child Health, University of Florence, Florence, Italy
Enrico Fainardi	Neuroradiology Unit, Department of Experimental and Clinical Biomedical Sciences, University of Florence, Florence, Italy
Stefano Chiti	Neuroradiology Unit, Department of Experimental and Clinical Biomedical Sciences, University of Florence, Florence, Italy.
Mattias Nilsson	Department of Clinical Neuroscience, Karolinska Institutet, Stockholm, Sweden
Henrik Viklund	Karolinska University Hospital Huddinge
Melissa Taheri Rydell	Department of Neurobiology, Care Sciences and Society; Center for Alzheimer Research, Division of Neurogeriatrics,, Bioclinicum, Karolinska Institutet, Solna, Sweden
Vesna Jelic	Department of Neurobiology, Care Sciences and Society; Division of Clinical Geriatrics, Karolinska Institutet, Stockholm, Sweden
Abbe Ullgren	Department of Neurobiology, Care Sciences and Society,; Center for Alzheimer Research, Division of Neurogeriatrics, Bioclinicum, Karolinska Institutet, Solna, Sweden
Elena Rodriguez-Vieitez	Department of Neurobiology, Care Sciences and Society,; Center for Alzheimer Research, Division of Neurogeriatrics, Bioclinicum, Karolinska Institutet, Solna, Sweden
Tobias Langheinrich	Division of Neuroscience and Experimental Psychology, Wolfson Molecular Imaging Centre, University of Manchester, Manchester, UK
Albert Lladó	Alzheimer’s disease and Other Cognitive Disorders Unit, Neurology Service, Hospital Clínic, Barcelona, Spain
Anna Antonell	Alzheimer’s disease and Other Cognitive Disorders Unit, Neurology Service, Hospital Clínic, Barcelona, Spain
Jaume Olives	Alzheimer’s disease and Other Cognitive Disorders Unit, Neurology Service, Hospital Clínic, Barcelona, Spain
Mircea Balasa	Alzheimer’s disease and Other Cognitive Disorders Unit, Neurology Service, Hospital Clínic, Barcelona, Spain
Nuria Bargalló	Imaging Diagnostic Center, Hospital Clínic, Barcelona, Spain
Sergi Borrego-Ecija	Alzheimer’s disease and Other Cognitive Disorders Unit, Neurology Service, Hospital Clínic, Barcelona, Spain
Ana Verdelho	Department of Neurosciences and Mental Health, Centro Hospitalar Lisboa Norte - Hospital de Santa Maria & Faculty of Medicine, University of Lisbon, Lisbon, Portugal
Carolina Maruta	Laboratory of Language Research, Centro de Estudos Egas Moniz, Faculty of Medicine, University of Lisbon, Lisbon, Portugal
Tiago Costa-Coelho	Faculty of Medicine, University of Lisbon, Lisbon, Portugal
Gabriel Miltenberger	Faculty of Medicine, University of Lisbon, Lisbon, Portugal
Frederico Simões do Couto	Faculdade de Medicina, Universidade Católica Portuguesa
Alazne Gabilondo	Cognitive Disorders Unit, Department of Neurology, Donostia University Hospital, San Sebastian, Gipuzkoa, Spain
Ioana Croitoru	Instituto de Investigación Sanitaria Biogipuzkoa, Neurosciences Area, Group of Neurodegenerative Diseases, San Sebastian, Spain.
Mikel Tainta	Instituto de Investigación Sanitaria Biogipuzkoa, Neurosciences Area, Group of Neurodegenerative Diseases, San Sebastian, Spain.
Myriam Barandiaran	Cognitive Disorders Unit, Department of Neurology, Donostia University Hospital, San Sebastian, Gipuzkoa, Spain
Patricia Alves	Instituto de Investigación Sanitaria Biogipuzkoa, Neurosciences Area, Group of Neurodegenerative Diseases, San Sebastian, Spain.
Benjamin Bender	Department of Diagnostic and Interventional Neuroradiology, University of Tübingen, Tübingen, Germany
David Mengel	Department of Neurodegenerative Diseases, Hertie-Institute for Clinical Brain Research and Center of Neurology, University of Tübingen, Tübingen, Germany
Lisa Graf	Department of Neurodegenerative Diseases, Hertie-Institute for Clinical Brain Research and Center of Neurology, University of Tübingen, Tübingen, Germany
Annick Vogels	Department of Human Genetics, KU Leuven, Leuven, Belgium
Mathieu Vandenbulcke	Geriatric Psychiatry Service, University Hospitals Leuven, Belgium; Neuropsychiatry, Department of Neurosciences, KU Leuven, Leuven, Belgium
Philip Van Damme	Neurology Service, University Hospitals Leuven, Belgium; Laboratory for Neurobiology, VIB-KU Leuven Centre for Brain Research, Leuven, Belgium
Rose Bruffaerts	Department of Biomedical Sciences, University of Antwerp, Antwerp, Belgium; Biomedical Research Institute, Hasselt University, 3500 Hasselt, Belgium
Koen Poesen	Laboratory for Molecular Neurobiomarker Research, KU Leuven, Leuven, Belgium
Pedro Rosa-Neto	Translational Neuroimaging Laboratory, McGill Centre for Studies in Aging, McGill University, Montreal, Québec, Canada
Maxime Montembault	Douglas Research Centre, Department of Psychiatry, McGill University, Montreal, Québec, Canada
Raffaella Lara Migliaccio	Sorbonne Université, Paris Brain Institute – Institut du Cerveau – ICM, Inserm U1127, CNRS UMR 7225, AP-HP - Hôpital Pitié-Salpêtrière, Paris, France
Ninon Burgos	Sorbonne Université, Paris Brain Institute – Institut du Cerveau – ICM, Inserm U1127, CNRS UMR 7225, AP-HP - Hôpital Pitié-Salpêtrière, Paris, France
Daisy Rinaldi	Sorbonne Université, Paris Brain Institute – Institut du Cerveau – ICM, Inserm U1127, CNRS UMR 7225, AP-HP - Hôpital Pitié-Salpêtrière, Paris, France
Catharina Prix	Neurologische Klinik, Ludwig-Maximilians-Universität München, Munich, Germany
Elisabeth Wlasich	Neurologische Klinik, Ludwig-Maximilians-Universität München, Munich, Germany
Olivia Wagemann	Neurologische Klinik, Ludwig-Maximilians-Universität München, Munich, Germany
Sonja Schönecker	Neurologische Klinik, Ludwig-Maximilians-Universität München, Munich, Germany
Alexander Maximilian Bernhardt	Neurologische Klinik, Ludwig-Maximilians-Universität München, Munich, Germany
Anna Stockbauer	Neurologische Klinik, Ludwig-Maximilians-Universität München, Munich, Germany
Jolina Lombardi	Department of Neurology, University of Ulm, Ulm
Sarah Anderl-Straub	Department of Neurology, University of Ulm, Ulm, Germany
Adeline Rollin	CHU, CNR-MAJ, Labex Distalz, LiCEND Lille, France
Gregory Kuchcinski	Univ Lille, France; Inserm 1172, Lille, France; CHU, CNR-MAJ, Labex Distalz, LiCEND Lille, France
Vincent Deramecourt	Univ Lille, France; Inserm 1172, Lille, France; CHU, CNR-MAJ, Labex Distalz, LiCEND Lille, France
João Durães	Neurology Department, Centro Hospitalar e Universitario de Coimbra, Coimbra, Portugal
Marisa Lima	Neurology Department, Centro Hospitalar e Universitario de Coimbra, Coimbra, Portugal
Maria João Leitão	Centre of Neurosciences and Cell Biology, Universidade de Coimbra, Coimbra, Portugal
Maria Rosario Almeida	Faculty of Medicine, University of Coimbra, Coimbra, Portugal
Miguel Tábuas-Pereira	Neurology Department, Centro Hospitalar e Universitario de Coimbra, Coimbra, Portugal; Faculty of Medicine, University of Coimbra, Coimbra, Portugal.
Sónia Afonso	Instituto Ciencias Nucleares Aplicadas a Saude, Universidade de Coimbra, Coimbra, Portugal
João Lemos	Faculty of Medicine, University of Coimbra, Coimbra, Portugal

## Abbreviations

ALS: Amyotrophic lateral sclerosis
AUC: Area under the curve
CDR® + NACC FTLD: CDR Dementia Staging Instrument + National Alzheimer’s Coordinating Centre Frontotemporal Lobar Degeneration component
C9orf72: Chromosome 9 open reading frame 72
CSF: Cerebrospinal fluid
DEBM: Discriminative event-based modelling
DPM: Disease progression model
FTLD: Frontotemporal lobar degeneration
GMM: Gaussian mixture modelling
GRN: Progranulin
MAPT: Microtubule-associated protein tau
MMSE: Mini Mental State Examination
NfL: Neurofilament light chain
pNfH: Phosphorylated neurofilament heavy chain
SuStaIn: Subtype and stage inference
