# Supplementary material for: Data-driven mapping of known and novel fluid biomarker progression profiles in genetic frontotemporal lobar degeneration
Source: Alzheimers Res Ther. 2026 Aug 11;18:243. doi: 10.1186/s13195-026-02159-5 (PMC13741760; doi:10.1186/s13195-026-02159-5)
Supplement: Supplementary file 1 [file 13195_2026_2159_MOESM1_ESM.docx]

**Supplemental material**

**Supplemental Table 1**

Summary metrics of the differential abundance analysis of our previous proteomics study^1^. To assess differences in protein abundance, we constructed linear regression models for each protein, with protein levels as the dependent variable and clinical group as the primary predictor, adjusting for age and sex. This analysis was performed for each genetic subgroup separately.

|  | *C9orf72* | | *GRN* | | *MAPT* | | Previously implicated in FTLD |
| --- | --- | --- | --- | --- | --- | --- | --- |
|  | log2FC | *P* | log2FC | *P* | log2FC | *P* |  |
| NfL | 1,50 | < 0,001 | 2,31 | < 0,001 | 1,96 | < 0,001 | ^2^ |
| TPM3 | 1,52 | < 0,001 | 2,08 | < 0,001 | 1,21 | < 0,001 | ^3^ |
| ELAVL4 | 0,77 | < 0,001 | 0,07 | 0,765 | 0,44 | 0,062 | ^4-6^ |
| CBLN4 | -0,57 | 0,003 | -0,73 | 0,002 | 0,14 | 0,626 | ^7,8^ |
| KRT18 | 0,73 | 0,005 | 0,29 | 0,36 | 0,64 | 0,072 | ^7^ |
| GRN | 0,17 | 0,995 | -1,01 | < 0,001 | 0,43 | 0,027 | ^9^ |
| SEMA3G | 0,18 | 0,995 | -1,08 | < 0,001 | 0,56 | 0,002 | ^10^ |
| SEZ6L | -0,03 | 0,995 | -0,15 | 0,001 | 0,12 | 0,206 | ^8^ |
| GFRA2 | -0,19 | 0,995 | -0,48 | 0,002 | 0,19 | 0,296 | ^7,8^ |
| NPTX2 | -0,17 | 0,995 | -0,68 | < 0,001 | -0,36 | 0,045 | ^11,12^ |
| MMP10 | 0,32 | 0,995 | 0,6 | 0,009 | 1,95 | < 0,001 | ^7,13^ |
| C1QA | 0,15 | 0,995 | -0,07 | 0,559 | 0,72 | < 0,001 | ^14^ |

**Supplemental Figure 1**

Volcano plots show the proteomic differences^1^ in symptomatic *C9orf72* (B), *GRN* (C) and *MAPT* (C) variant carriers compared to non-carriers. These plots are based on linear regression models for each protein, with protein levels as the dependent variable and clinical group as the primary predictor, adjusting for age and sex.

**Supplemental Figure 2**

Heatmap illustrating the protein abundance fold changes^1^ for the top 10 significant proteins in symptomatic *C9orf72*, *GRN* and ***MAPT*** carriers compared to non-carriers. The log_2_ fold change in protein abundance between groups is color-coded, with higher abundance shown in red and lower abundance in blue.

**Supplemental Figure 3. Distributions of selected proteins**

Gaussian Mixture Model (GMM) distributions of selected protein values in each genetic subgroup: (A) *C9orf72*, (B) *GRN*, and (C) *MAPT*. Histogram bins represent non-carriers (blue), presymptomatic carriers (orange), and symptomatic carriers (red). The blue dotted curve indicates the Gaussian distribution of normal protein values estimated from non-carriers, while the red dotted curve represents the distribution of abnormal values, estimated by GMM based on data from both presymptomatic and symptomatic carriers. Black curves show the total estimated protein distribution for each subgroup.

**Supplemental Figure 4. Estimation of protein** **orderings in the three genetic subgroups.**

The event-center variance diagrams ((A) *C9orf72*, (B) *GRN*, and (C) *MAPT*) show the standard errors of the estimated event centers. The protein ordering is based on their positions in the full dataset without bootstrap resampling, reflecting the mean estimated order.

**Supplemental Figure 5**. **Differences in relative protein abundance between symptomatic *C9orf72* carriers with an estimated disease stage < 0.5 and ≥ 0.5**

Boxplots showing differences in relative protein abundance among symptomatic *C9orf72* carriers, stratified by estimated disease stage (< 0.5 vs. ≥ 0.5). Protein abundance between the two groups was compared using a *t* test. * *P* < 0.05, ** *P* < 0.01

**Supplemental Figure 6. Correlation between MMSE and estimated disease stages**

Scatterplot displaying the correlation between MMSE and the estimated disease stages in each genetic subgroup: (A) *C9orf72*, (B) *GRN*, and (C) *MAPT*. Horizontal jitter was added to MMSE scores for visualization purposes only to reduce overlap between observations with identical scores; the original values were used for all statistical analyses.

**Supplemental Figure 7. Correlation between CDR® plus NACC FTLD sum of boxes and estimated disease stages**

Scatterplot displaying the correlation between CDR® plus NACC FTLD sum of boxes and estimated disease stages in each genetic subgroup: (A) *C9orf72*, (B) *GRN*, and (C) *MAPT*. Horizontal jitter was added to CDR® plus NACC FTLD sum of boxes scores for visualization purposes only to reduce overlap between observations with identical scores; the original values were used for all statistical analyses.

**References**

1.	De Houwer JFH, Dopper EG, van Buuren R, et al. Distinct proteomic CSF profiles in genetic frontotemporal lobar degeneration. *Brain*. Dec 4 2025;

2.	Meeter LH, Dopper EG, Jiskoot LC, et al. Neurofilament light chain: a biomarker for genetic frontotemporal dementia. *Ann Clin Transl Neurol*. Aug 2016;3(8):623-36.

3.	Pesämaa I, Müller SA, Robinson S, et al. A microglial activity state biomarker panel differentiates FTD-granulin and Alzheimer's disease patients from controls. *Mol Neurodegener*. Sep 29 2023;18(1):70.

4.	De Santis R, Alfano V, de Turris V, et al. Mutant FUS and ELAVL4 (HuD) Aberrant Crosstalk in Amyotrophic Lateral Sclerosis. *Cell Rep*. Jun 25 2019;27(13):3818-3831 e5.

5.	Bowles KR, Silva MC, Whitney K, et al. ELAVL4, splicing, and glutamatergic dysfunction precede neuron loss in MAPT mutation cerebral organoids. *Cell*. Aug 19 2021;184(17):4547-4563 e17.

6.	Silvestri B, Mochi M, Mawrie D, et al. HuD impairs neuromuscular junctions and induces apoptosis in human iPSC and Drosophila ALS models. *Nat Commun*. Nov 7 2024;15(1):9618.

7.	Saloner R, Staffaroni AM, Dammer EB, et al. Large-scale network analysis of the cerebrospinal fluid proteome identifies molecular signatures of frontotemporal lobar degeneration. *Nat Aging*. Jun 2025;5(6):1143-1158.

8.	Sogorb-Esteve A, Weiner S, Simrén J, et al. Proteomic analysis reveals distinct cerebrospinal fluid signatures across genetic frontotemporal dementia subtypes. *Sci Transl Med*. Feb 5 2025;17(784):eadm9654.

9.	Ghidoni R, Benussi L, Glionna M, Franzoni M, Binetti G. Low plasma progranulin levels predict progranulin mutations in frontotemporal lobar degeneration. *Neurology*. Oct 14 2008;71(16):1235-9.

10.	Gerrits E, Giannini LAA, Brouwer N, et al. Neurovascular dysfunction in GRN-associated frontotemporal dementia identified by single-nucleus RNA sequencing of human cerebral cortex. *Nat Neurosci*. Aug 2022;25(8):1034-1048.

11.	van der Ende EL, Meeter LH, Stingl C, et al. Novel CSF biomarkers in genetic frontotemporal dementia identified by proteomics. *Ann Clin Transl Neurol*. Apr 2019;6(4):698-707.

12.	van der Ende EL, Xiao M, Xu D, et al. Neuronal pentraxin 2: a synapse-derived CSF biomarker in genetic frontotemporal dementia. *J Neurol Neurosurg Psychiatry*. Jun 2020;91(6):612-621.

13.	Hok AHYS, Vermunt L, Peeters CFW, et al. Large-scale CSF proteome profiling identifies biomarkers for accurate diagnosis of frontotemporal dementia. *Mol Neurodegener*. Aug 27 2025;20(1):93.

14.	van der Ende EL, Heller C, Sogorb-Esteve A, et al. Elevated CSF and plasma complement proteins in genetic frontotemporal dementia: results from the GENFI study. *Journal of Neuroinflammation*. Sep 5 2022;19(1):217.
